# Carbon Nanotube Membranes for use in the Transdermal Treatment of Nicotine Addiction and Opioid Withdrawal Symptoms

**DOI:** 10.4137/sart.s1050

**Published:** 2009-03-18

**Authors:** Caroline L. Strasinger, Nicole N. Scheff, Ji Wu, Bruce J. Hinds, Audra L. Stinchcomb

**Affiliations:** 1 Department of Pharmaceutical Sciences, University of Kentucky, Lexington, KY, U.S.A.; 2 Department of Chemical and Materials Engineering, University of Kentucky, Lexington, KY, U.S.A.

**Keywords:** transdermal delivery, variable rate, CNT, nicotine, clonidine

## Abstract

Transdermal systems are attractive methods of drug administration specifically when treating patients for drug addiction. Current systems however are deficient in therapies that allow variable flux values of drug, such as nicotine for smoking cessation or complex dosing regimens using clonidine when treating opioid withdrawal symptoms. Through the use of functionalized carbon nanotube (CNT) membranes, drug delivery to the skin can be controlled by applying a small electrical bias to create a programmable drug delivery system. Clearly, a transdermal patch system that can be tailored to an individual’s needs will increase patient compliance as well as provide much more efficient therapy. The purpose of this paper is to discuss the applicability of using carbon nanotube membranes in transdermal systems for treatment of drug abuse.

## Introduction

Drug abuse, whether alcohol, tobacco, prescription or illicit, continues to burden society, economics, medical care systems, and the family dynamic. The World Health Organization (WHO) estimates that globally 185 million people are illicit drug users and an astounding 1.3 billion individuals are smokers.^1^ Smoking related deaths, estimated at nearly 5 million worldwide, continue to be the leading preventable cause of death. Additionally, in the United States alone, $167 billion in annual health-related economic loss can be attributed to smoking.^2^ Although there are roughly only 200,000 deaths reported to be directly associated with illicit drug use, the detriment to family and society, and the economic costs associated with crime, law enforcement, medical care, and drug treatment and rehabilitation, are immense.^3^,^4^ While current treatments for cigarette smoking and opioid addiction do aid in successful abstinence from the individual’s vice, there is great need and room for improvement. The best of smoking cessation therapies reports a mere 19% success after one year, while only 50% of individuals who enter clinics for opioid addiction remain for the duration of the treatment, of which 65% return to using within 4 weeks.^3^,^5^ Clearly, a therapy that better controls cravings or abates withdrawal symptoms to decrease the likelihood of relapse is needed. Carbon nanotube (CNT) membranes present the opportunity to create a transdermal patch that can vary its rate of delivery throughout its application to the skin to attain therapeutic plasma levels and plasma profiles of a specific drug. The purpose of this report is to discuss the use of carbon nanotube membranes in a transdermal system to aid in abstinence from cigarettes and withdrawal from opioids.

## Nicotine and Clonidine as Therapies

Conventional nicotine replacement therapy (NRT) has been on the market since the early 1990s. Continuous research and development of the therapy has expanded the delivery from early reservoir transdermal patches to matrix systems, gums, inhalers, lozenges, and nasal sprays.^6^ As the name implies, NRT works to replace nicotine, the agent in cigarettes responsible for dependence, with a pure pharmaceutical source, thereby eliminating the toxic chemicals found in tobacco smoke from entering the body. Furthermore, NRT prevents withdrawal symptoms which induce the desire to smoke, thereby increasing a smoker’s ability to put up with the environmental and social cues associated with smoking. Though current NRTs do aid in smoking cessation when compared to individuals who try to quit using non-pharmacological treatment or are given placebos, the success rate is still an unsatisfactory 17%.^3^ A representative daily nicotine plasma profile of a heavy smoker can be seen in Figure 1a. It consists of a series of peaks and troughs spurred by the smoking of each cigarette with rapid, less than five minutes, attainment of the maximum nicotine level. Slight accumulation can be seen over the course of the day, but the high spike of nicotine is always obtained with the smoking of a new cigarette.^7^ NRT’s low success rate is primarily due to the fact that no current system can actually mimic the rapid attainment of high nicotine plasma levels associated with smoking a cigarette.^3^,^8^ Nasal spray offers the fastest time of delivery to its maximum concentration, but does not reach the level required to suppress a nicotine craving of a smoker, and like the gum, lozenge or inhaler it requires multiple doses throughout the day which often leads to issues with patient compliance. The current transdermal patch is a once daily application, however it cannot safely deliver the craving sustaining dose, nor can the system deliver it in a rapid manner, mimicking the smoking plasma profile.^6^ Clearly, a delivery system that can rapidly and safely deliver nicotine at a level equal to that of a cigarette, followed by a return to baseline levels, and then later be reactivated when a craving occurs, would be beneficial to someone attempting to abstain from smoking. Furthermore, a system that can be tailored to an individual’s smoking schedule would provide a more efficacious treatment than a standard “one size fits all” therapy.

Similarly, a multiple rate delivery system would be beneficial to patients and medical staff when treating opiate withdrawal symptoms using clonidine. Currently, clonidine is used in rehabilitation clinics to mitigate the symptoms of opiate withdrawal including rhinorrhea, lacrimation, perspiration, piloerection, gastrointestinal complaints, nausea, yawning, sneezing, muscle pain, tachycardia and hypertension.^4^ Clonidine is a partial pre- and postsynaptic alpha-adrenoreceptor agonist that produces a reduction in peripheral sympathetic outflow. Patients experience acute withdrawal symptoms between 6 to 12 hours after opioid abstinence with peak effects being felt between 36 to 72 hours and gradual abatement over 5 to 10 days.^3^,^9^ For this reason patients are typically given multiple oral clonidine doses, or in a few cases a combination of transdermal and oral drug, per day during treatment.^4^,^5^ A typical five day oral treatment profile can be seen in Figure 1b. Clearly, repetitive dosing with variable amounts each day puts a strain on the patient and clinical staff. Patients who are experiencing withdrawal are likely irritable and unreceptive to medical staff who must administer the multiple doses of a drug per day. Additionally, patients who suffer nausea and gastrointestinal problems during withdrawal experience difficulties in maintaining proper doses due to vomiting. A transdermal system that can manipulate its delivery rate to provide the required dose on specific days during a typical clinic stay would not only eliminate the physiological dosing issues, but also ease the burden of administering, monitoring, and tailoring a drug regimen to each individual patient.

Transdermal patches tend to be one of the most desired therapies by patients and health care providers, not only for easy and pain-free administration, but also for several pharmacokinetic and pharmacodynamic benefits.^10^,^11^ Traditional transdermal delivery allows one time application to constantly administer drug over a given time, thereby eliminating the hassle and difficulties associated with oral or intravenous maintenance multiple dosing. This “worry free” administration increases patient compliance, which is essential when trying to combat drug addiction. Additionally, once a drug passes through the upper echelons of the skin, it is directly taken into systemic circulation by the highly vascularized dermal layers. This affords the opportunity to lower the amount of drug required at the administration site, because of the avoidance of first pass metabolism. Not only is this more cost effective, but also safer for the patient as high levels of compounds can increase the chance for toxicity. Furthermore, as mentioned previously, gastrointestinal complications can lead to difficult and improper oral dosing when treating patients experiencing opioid withdrawal symptoms. Such symptoms would not hamper therapeutic regimen designs when delivering the desired pharmaceutical agent transdermally. Despite the attractiveness of the transdermal route, one major shortcoming exists in efficaciously treating smoking and opioid addiction with currently available patches; none of the current marketable transdermal patches can deliver the required variable dosing rate of drug to effectively treat these addictions in a single, self-contained system. The use of CNT membranes in a transdermal system offers a feasible solution to this variable rate drug delivery predicament.

## Carbon Nanotube Membranes

The demand for development of highly functionalized chemical screening, separating, purifying and delivery devices has risen in the past several decades as vast advances in the areas of biology, chemistry, electronics and medicine continue to be made.^12^ Nanoporous membranes consisting of aligned, multi-walled CNTs in a polymer substrate, possessing distinctive properties have demonstrated their applicability in the areas of electronic devices, chemical separation, biosensors, salt water desalination and purification, and most recently, in the area of drug delivery. More specifically, three significant properties unique to CNT membranes that can be utilized to conquer the difficulties of a variable rate drug therapy are 1) extremely fast fluid flow due to graphite planes and large van der Waals distance,^13^ 2) well defined geometry of ligands at pore entrance resulting from membrane synthesis,^14^ and 3) the concentration of an electric field at the tips of CNTs due to their electrically conductive properties while surrounded by an insulating polymer support.^15^ Synthesis of these membranes has been described previously,^16^ but briefly carbon nanotube arrays are grown by chemical vapor deposition (CVD) on a quartz substrate using specific metal catalysts to control the diameter of nanotubes. The spaces between the individual tubes are then embedded with a polymer substrate and the quartz support etched off using hydrofluoric acid. H_2_O plasma oxidation is utilized to remove excess polymer on the surface of the film as well as the initial growth catalyst particle. This process results in a robust membrane with open-ended CNTs possessing carboxylic functionality at the tips. These tips can be further functionalized with various molecules using carbodiimide chemistry to create charged molecular chains that, with proper electrical influence, will work as a gate to chemical transport through the nanotubes.^15^ More specifically and as seen in Figure 2, by applying a small electrical bias (<50 mV) to the membrane, the end groups, which are negatively charged, are either attracted to the nanotubes themselves causing blockage of the pore and thus no transport, or repelled from one another and surrounding nanotubes causing the functional group to stand on end allowing fluid passage and drug delivery through the pore. By incorporating this voltage gated membrane into a transdermal system (Fig. 3a), programmable controlled dosing either by physician or patient is attainable.

Recall that the major set back in current NRT therapies is the inability to mimic the actual pulsatile nicotine delivery profile and resultant peak plasma levels of a smoker. With the ability of a patch to deliver a high dose of nicotine and then safely shut off to return to baseline plasma levels throughout the day, a smoker would be able to satisfy their nicotine craving and thereby increase their chances of becoming smoking independent. Furthermore, these patches can be patient controlled through a push activated button pressed at the initiation of a patient’s craving, which would apply the electrical bias to the membrane allowing drug flow to the skin, eliminating patient to patient tailoring issues seen with conventional therapies. As with all drug addiction and subsequent pharmacological replacement treatment, the patient must be motivated to quit to truly gain independence from whatever drug one is dependent upon.

Similarly, the multiple and variable dosages that are utilized to properly treat opioid withdrawal symptoms can be simplified to a one time application and computer chip controlled transdermal flux change system. Using multiple membranes within the patch will allow for a therapy that is patient tailorable and highly desirable for both patient and medical staff. Using the setup seen in 3b, the typical clonidine dosing profile previously discussed can be achieved by activating one side of the patch the first and fourth day, activating both membranes the second and third day for the highest transdermal flux, and by activating only the second membrane the fifth day to taper down the dose.

As alluded to earlier, abusers going through withdrawal may experience increased sweating, variable circulation and based on the degree of withdrawal, craving, or intoxication may be quite different from patient to patient.^17^ This variability in skin conductance could introduce problems in traditional transdermal delivery; however one of the main advantages of the CNT membrane system is its increased dosing flexibility. More specifically, the system cannot only be tailored to an individual patient’s needs, but also possesses the added bene fit of flexibility in drug delivery as conditions change over the course of treatment. Because of the high doses of nicotine required to effectively treat smoking addiction with this nanotube system and the fact that the system will be patient activated, the potential for lethal overdose is a possibility without a proper design. For this reason the patch will require a multi-use lockout system; the first facet to automatically shut off the patch when the proper nicotine amount is delivered, the second to prevent the addicted individual from activating the patch too often and delivering too much nicotine in a short time period. The proper amount of nicotine refers to the required amount of drug to be delivered transdermally that corresponds to the nicotine plasma levels seen immediately following the smoking of a cigarette. Additionally, as discussed, this patch setup will involve the use of a small electric current to deliver the drug. Currently marketed transdermal iontophoresis systems also use electric current to deliver drug. However, unlike iontophoresis, the electrodes used to deliver the bias are never in contact with the skin, nor is the voltage required as high; 50 mV for the CNT system compared to 10 V for iontophoresis.^15^,^18^ For these reasons the skin irritation and lesions that are often seen with the iontophoretic set up are not expected when using CNT membranes in a transdermal patch.^19^

The use of CNT membranes in transdermal therapy is fairly cost effective as the membranes themselves can be fashioned for as little as $0.60/m^2^. The bulk of the cost of this transdermal set up will come from cost of drug and the bias control system. The cost of drug will obviously be comparable to that of current nicotine and clonidine transdermal therapies. The voltage required to activate and deactivate the system is no more than that which can be supplied by a watch battery, and the mechanics of the system, electrodes to provide the bias, is comparable to that of the new patient activated iontophoteric fentanyl delivery system marketed as IONSYS^TM^.^20^ Additionally, the voltage system would be able to be reused, making the drug the only continuous expense throughout the therapy.

## Therapeutic Feasibility

Despite the simplicity of the system, it was still essential to demonstrate that the skin alone could permit the required maximum transdermal flux, proper rate changes, and especially in the case of nicotine delivery, do so with minimal lag time. A series of proof-of-concept *in vitro* diffusion studies were conducted on excised human abdominal skin in order to determine if the three parameters discussed were feasible for each drug. The average smoker has a steady-state nicotine plasma concentration of about 10–20 ng/ml with peak levels reaching approximately 54 ng/ml throughout the day with each cigarette.^7^ Using equation 1 in which J is the steady-state transdermal flux, *C_ss_* is the steady-state plasma concentration, Cl is the total body clearance of nicotine, and A is the diffusional area (7 cm^2^), these plasma levels translate to a required transdermal flux of 0.8 μmol/cm^2^/h and 4 μmol/cm^2^/h for steady-state and peak nicotine concentrations, respectively. For a traditional five-day opioid withdrawal symptom treatment regimen using clonidine, the first and fourth days require a total of 0.2 mg per day of oral clonidine, days two and three a total of 0.3 mg per day, and the fifth day 0.1 mg of oral clonidine as seen in Figure 1b.^5^ Using a patch size of 10 cm^2^, 100% bioavailability,^21^ and each day’s oral dose over a 24 hour period, effective transdermal fluxes of 3.5 nmol/cm^2^/h, 5.4 nmol/cm^2^/h, and 1.7 nmol/cm^2^/h can be estimated.

(1) $$J=\frac{C_{ss}Cl}{A}$$

Human skin from abdominoplasty surgery was obtained from the National Cancer Institute’s Cooperative Human Tissue Network (CHTN). The samples were dermatomed immediately upon arrival to a thickness of approximately 200 μm and frozen at −20 °C. On the day of the experiment, the skin was thawed and immediately used for the diffusion studies. The abdominal skin was loaded into a cell of a PermeGear^®^ In-Line Diffusion apparatus (Bethlehem, PA, U.S.A) and charged with 250 μl of desired drug solution, and temperature was maintained at 35 °C (skin surface temperature) by a circulating water bath.^22^ Solutions of 0.9% saline for nicotine and 0.9% saline with 10% ethanol for clonidine were prepared for use as the receiver solutions for diffusion. Multiple donor concentrations were used in order to simulate a CNT change in drug supply to the skin surface to determine whether a flux change would be observed through the skin, and to see its associated transition (lag) time for rate change. For example, after it was determined that a nicotine donor concentration of 500 mg/ml would produce the desired 4 μmol/cm^2^/h flux, this donor solution was applied to the skin and the receiver solution was sampled over 30 minutes. Next, the donor solution was removed and the skin and diffusion cell donor chamber quickly rinsed with water to remove any residual nicotine on the surface, and a much lower donor solution concentration of 50 mg/ml was used in order to observe a flux change and lag time to flux change. After another 30 minutes of receiver solution sampling, the skin and cell chamber were washed again and a donor solution of 500 mg/ml was reapplied to the skin, and the receiver solution sampled again. This process mimics the activation of the carbon nanotube membrane to allow more drug flow, followed by deactivation and then reactivation, much like a person smoking two cigarettes over an hour and a half period. Transition times were observed and results can be seen in Table 1. The term transition time for this work refers to the time it takes between a change in drug rate supply to reach the new desired steady-state concentration in the receiver solution.

**Figure 4:**
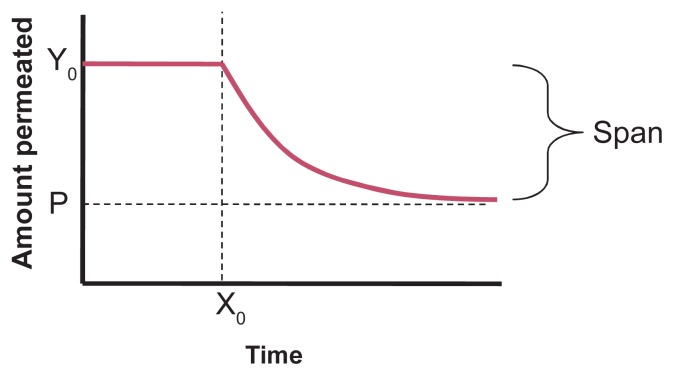
Associated figure for mathematical model equation 2.

It was also important to investigate the rate of drug delivery out of the skin after a donor concentration was removed, in other words the depletion of nicotine from the skin after the patch is turned off. The residual drug amount that is in the skin after the patch is shut off or removed will enter systemic circulation and must be taken into consideration when determining the proper time to allot between patient initiated activation. For this study, varying donor solution concentrations (200, 300, 400, 500 mg/ml) were applied to the skin long enough to reach steady state, and then removed and the cell chamber and skin briefly washed with water. Sampling of the receiver solution was continued for approximately two hours with no donor solution on the skin in order to observe nicotine depletion. These studies resulted in the model seen in Equation 2, where the variables correspond to Figure 4 and *k* is the time constant which was 0.088 ± 0.0092 min^−1^ (Graphpad Prism®, La Jolla, CA, U.S.A) and independent of donor concentration (p > 0.05) (one-way ANOVA, SigmaStat3.5^©^, San Jose, CA, U.S.A). This indicated that the skin will not have a significant depot effect once the patch is removed or the system is completely shut off and that proper lockout periods can be programmed based on this equation.

(2) $$Y=P+(Y_{0}-P)\times e^{-k(X-X_{e})}$$

Clonidine also had to be investigated to ensure that the proper therapeutic levels of drug could be reached when delivered transdermally, in addition to if the skin responds to a change in donor concentration allowing a subsequent change in transdermal flux. A similar process of applying a donor solution followed by a brief washing of the skin and diffusion cell donor compartment was repeated, except that this time each donor solution remained on the skin for 24 hours thereby more closely representing therapeutic clonidine delivery. The required therapeutic flux values for each of the different doses were achieved and can also be seen in Table 1, but it should be noted a short transition time between doses is not as important as it is with nicotine, as tapering doses over the 5–10 day treatment are currently used in the clinical setting.

## Future CNT Membrane Application

It is easy to envision the use of the CNT membrane in therapies not discussed here. Its applicability is wide and the ability to change the rate of delivery will prove highly beneficial in many therapeutic areas. Any skin permeable drug is suitable for CNT membrane delivery as the membrane is not rate limiting in the way the skin can be based on size of the molecule or lipophilicity of the drug. CNT membranes do not inhibit the use of transdermal techniques to increase the breadth of drugs available for use in transdermal systems such as prodrugs and codrugs, or the use of microneedles prior to patch application.^23^–^26^ Using these techniques in conjunction with CNT membranes would not only allow a wider range of drugs to be delivered, but also provide more flexibility in programmable rates. By increasing the permeation through the skin, the range of high to low flux through the membrane is increased allowing finer patient to patient tailoring. Additionally, a multiple drug delivery system could be developed in which two drug reservoirs of different content and two separate CNT membranes are utilized. This would allow for simultaneous delivery of two drugs at varying rates, or for transfer between medications without removal and application of a new transdermal system. Obviously, size and cost of the device would increase as the system becomes more advanced, however the benefits of variable delivery would outweigh these disadvantages in some therapeutic applications.

## Conclusions

Carbon nanotube membranes present a feasible solution to pitfalls in current smoking cessation and opioid withdrawal symptom treatments. Therapeutic transdermal fluxes can be achieved and the skin responds well to changes in donor concentrations indicating that the skin will not be a limiting factor when bias is applied to change the flux from the patch. This novel method to create a rate changing drug delivery device will not only increase patient compliance because of the multiple benefits of using the transdermal route, but also provide more effective therapy and improve the quality of life for many addicted individuals.

## Figures and Tables

**Figure 1a:**
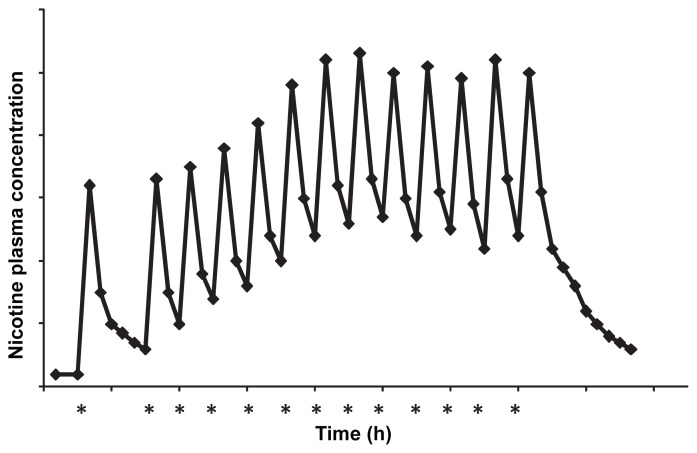
Representative nicotine plasma profile of a cigarette smoker. Asterisks indicate the smoking of a cigarette.

**Figure 1b:**
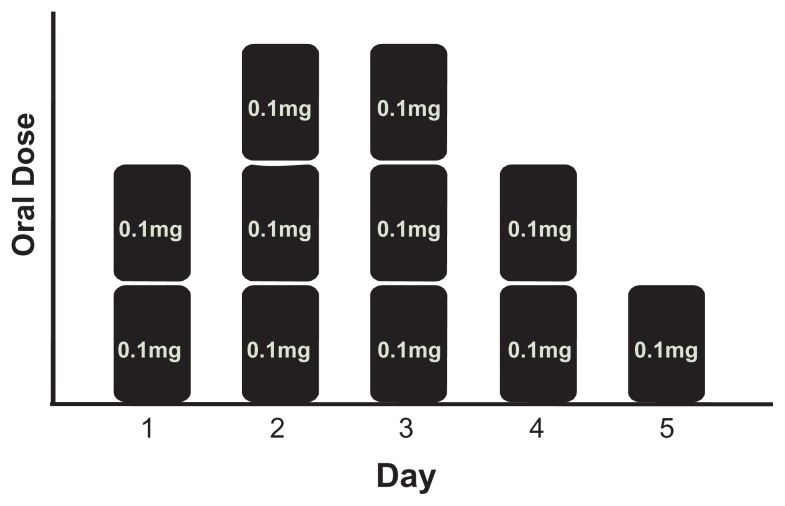
Representative 5 day opioid withdrawal symptom treatment with clonidine dosing scheme. On day one two 0.1 mg doses are given for a total of 0.2 mg of clonidine, on day two three doses are given and so on.

**Figure 2a:**
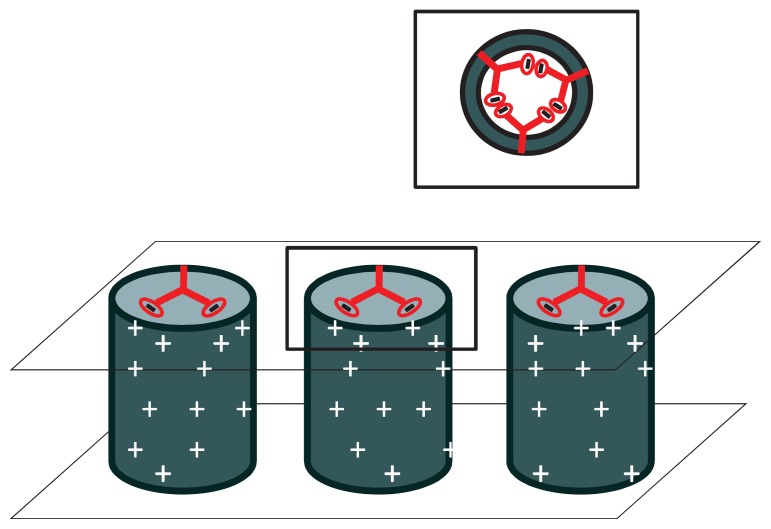
No or limited drug diffusion (depending on the size of the attached dendrimer) occurs when a positive bias is applied to the membrane. Each nanotube has multiple dendrimers attached to opening as shown in the inset, for simplification purposes only one is shown in the larger figures.

**Figure 2b:**
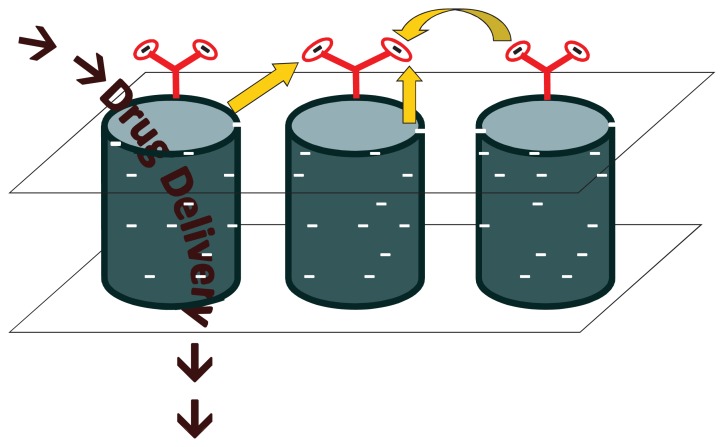
Drug passes through the nanotubes when a negative bias is applied. The dendrimers remain upright opening the tube because of the surrounding negative charges as shown by the arrows.

**Figure 3a:**
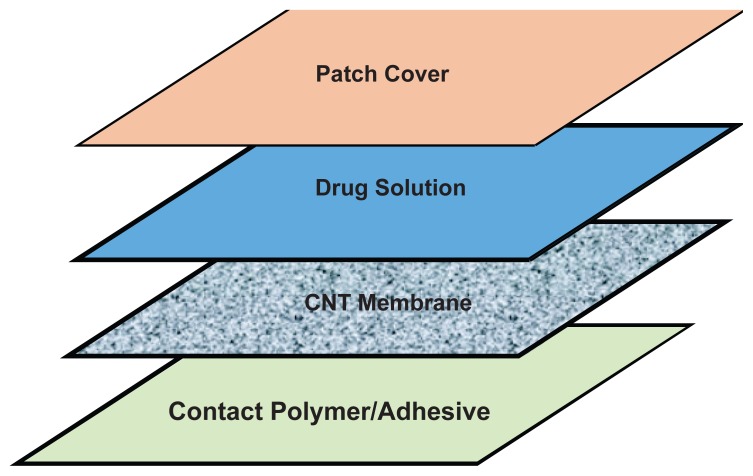
Schematic of patch layering using one CNT membrane to produce two different transdermal fluxes.

**Figure 3b:**
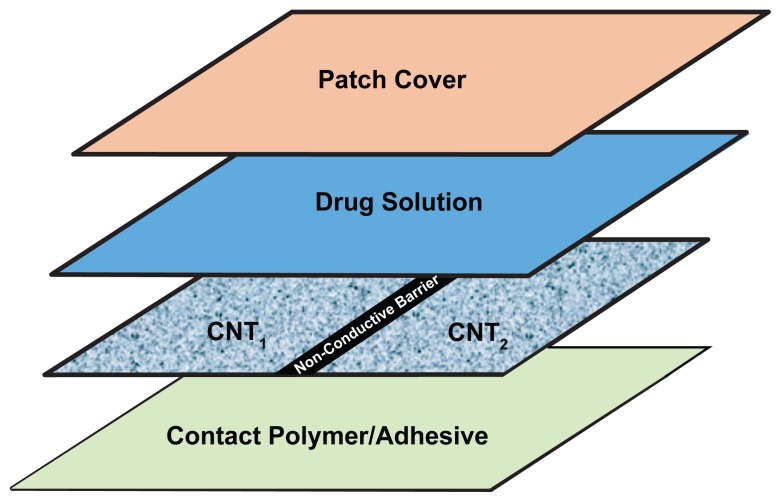
Schematic of patch layering using two CNT membranes to produce three different transdermal fluxes.

**Table 1:** Therapeutic transdermal fluxes and transition times for nicotine and clonidine (n = 4).

	Concentration (mg/ml)	Transdermal flux (μmol/cm^2^/h)	Transition time (min)
Nicotine	500	4.27 ± 0.22	4.7 ± 0.4
	50	1.48 ± 0.22	0
	500	4.23 ± 0.25	5.5 ± 0.5
Clonidine	0.225	0.0037 ± 0.00100	322 ± 59*
	0.325	0.0053 ± 0.00068	N/A
	0.160	0.0021 ± 0.00096	N/A

* Initial lag time for the first dose.
